# The transcription factor Ets21C drives tumor growth by cooperating with AP-1

**DOI:** 10.1038/srep34725

**Published:** 2016-10-07

**Authors:** Janine Toggweiler, Maria Willecke, Konrad Basler

**Affiliations:** 1 Institute of Molecular Life Sciences, University of Zurich, Zurich, Switzerland

## Abstract

Tumorigenesis is driven by genetic alterations that perturb the signaling networks regulating proliferation or cell death. In order to block tumor growth, one has to precisely know how these signaling pathways function and interplay. Here, we identified the transcription factor Ets21C as a pivotal regulator of tumor growth and propose a new model of how Ets21C could affect this process. We demonstrate that a depletion of Ets21C strongly suppressed tumor growth while ectopic expression of Ets21C further increased tumor size. We confirm that Ets21C expression is regulated by the JNK pathway and show that Ets21C acts via a positive feed-forward mechanism to induce a specific set of target genes that is critical for tumor growth. These genes are known downstream targets of the JNK pathway and we demonstrate that their expression not only depends on the transcription factor AP-1, but also on Ets21C suggesting a cooperative transcriptional activation mechanism. Taken together we show that Ets21C is a crucial player in regulating the transcriptional program of the JNK pathway and enhances our understanding of the mechanisms that govern neoplastic growth.

The transformation of a healthy cell into a malignant derivative is a complex process that involves various genetic alterations. Such mutations often result in perturbations of signaling pathways and allow cancer cells to acquire the capability to produce their own growth factors, to become insensitive to anti-growth signals, to evade apoptosis and to migrate out of their tissue of origin - features considered as the hallmarks of cancer that lead to uncontrolled growth^1^,^2^. To develop strategies against cancer cells, it is important to know how different mutations cooperate and how signaling networks function and interplay.

Tumor models have been established in *Drosophila* that are based on defined genetic modifications and mimic key hallmarks of human cancers^3^. In one of these models, tumors are generated by concomitantly activating Ras signaling (Ras^V12^) and downregulating the polarity determinant Discs-large (Dlg). The combination of loss of polarity and signaling downstream of Ras leads to the formation of large, neoplastic tumors^4^. Dlg is required to establish and maintain apical-basal polarity and forms a complex with two other proteins, Scribbled (Scrib) and Lethal giant larvae (Lgl)^5^,^6^. Loss of polarity is thought to activate the JNK pathway through association of apical polarity determinants with the TNF receptor Grindelwald (Grnd)^7^. In this context, the JNK pathway is a key player in driving tumor growth and malignancy. It activates the expression of Matrix metalloproteinase 1 (Mmp1) that degrades extracellular matrix proteins, thereby allowing tumor cells to become mobile and to migrate out of their tissue of origin^8^. Furthermore, JNK stimulates JAK/STAT signaling by upregulating the expression of the Unpaired (Upd) cytokines, Upd1, Upd2 and Upd3. *Ras*^*V12*^ *dlg*^*RNAi*^ tumors are therefore a good system to study Ras signaling and the JNK pathway or the interplay between the two.

In order to unearth new regulators of tumor growth, we have conducted a microarray-based screen and identified the transcription factor Ets21C as a crucial regulator of tumor growth. As the function of Ets21C was only poorly characterized at this time, we sought to investigate its role in tumorigenesis in more detail. For this purpose we addressed the following questions: Why is Ets21C upregulated in *Ras*^*V12*^ *dlg*^*RNAi*^ tumors? Which target genes are activated by Ets21C that can explain its effects on tumor growth? We show that changes in Ets21C levels critically affect tumor growth and that Ets21C expression is regulated via the JNK pathway. Furthermore, we find that Ets21C induces specific downstream effectors of the JNK pathway that are known to drive tumor growth. The transcriptional activation depends on the DNA binding domain of Ets21C and likely on a cooperation with the AP-1 transcription factor, revealing Ets21C as a key nuclear effector of the JNK pathway.

## Results

### *Ras* ^*V12*^ *dlg* ^*RNAi*^ tumors require Ets21C for growth

**Figure 1:**
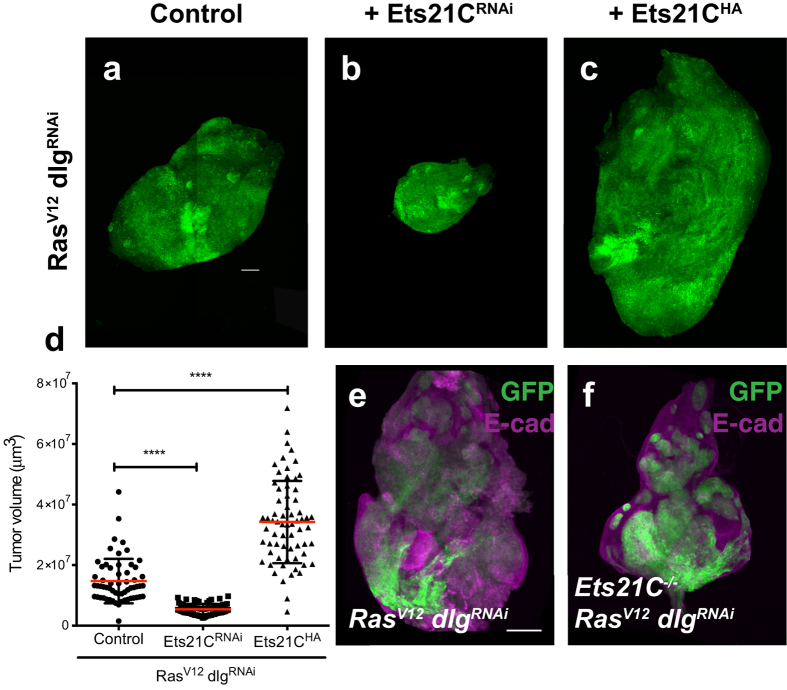
Ets21C is required for *Ras*^*V12*^ *dlg*^*RNAi*^ tumors. Confocal images of (**a–c**) imaginal disc tumors expressing different UAS-transgenes under the control of eye specific Gal4: Flp recombinase is driven by the *eyeless* (*ey*) promoter, and causes activation of the act > CD2 > Gal4 transgene. Recombined cells are marked by expression of a *UAS-GFP* transgene (green). The discs co-express the following transgenes: (**a**) *Ras*^*V12*^*, dlg*^*RNAi*^, (**b**) *Ras*^*V12*^*, dlg*^*RNAi*^*, Ets21C*^*RNAi*^, (**c**) *Ras*^*V12*^*, dlg*^*RNAi*^*, Ets21C*^*HA*^. (**d**) Quantification of tumor volume for the genotypes indicated (Mann-Whitney test ****p < 0.0001). Error bars indicate SD. (**a**,**c**) are composites of several images to display the whole tumor size. (**e**,**f**) *ey-FLP* induced MARCM clones^34^ that are positively labeled with GFP (green). Additionally, discs are stained for E-cadherin (E-cad) to visualize cell outlines (magenta). (**e**) *Ras*^*V12*^ *dlg*^*RNAi*^ expressed in wild-type clones, (**f**) *Ras*^*V12*^ *dlg*^*RNAi*^ expressed in clones homozygous mutant for *Ets21C*. Scale bar: 100 μm.

To identify new components that regulate tumor growth, we used our recently developed *Ras*^*V12*^ *dlg*^*RNAi*^ tumor model that can assess effects of additional transgenes in only one generation^4^. We tested a subset of genes that we have found upregulated in *Ras*^*V12*^ *dlg*^*RNAi*^ tumors by RNAi and screened for modifications of the tumor phenotype. The details of the screen will be published elsewhere. An RNAi targeting the *Ets21C* (*CG2914*) transcript acted as a strong suppressor of tumor growth (Fig. 1). Tumors depleted of Ets21C are reduced to half the size of control tumors (Fig. 1a,b,d). To exclude the possibility that the growth suppressing effects are caused by off-target activities of the RNAi transgene, we created a null allele of the *Ets21C* gene by inducing a deletion that removed a major part of the gene. As the deletion is homozygous lethal, we used the MARCM system to generate clones homozygous mutant for *Ets21C* and expressing *Ras*^*V12*^ and *dlg*^*RNAi*^. Similar to the effects of the *Ets21C*^*RNAi*^, deleting *Ets21C* led to a reduction of tumor size and to a significant restoration of disc morphology (Fig. 1e,f). Importantly, the size reduction is specific for the tumor as *Ets21C* mutant clones in a wild-type background grow normally (Supplementary Fig. 1a,b).

Since depletion of Ets21C had tumor growth suppressing effects, we wondered whether higher levels of Ets21C would promote growth of *Ras*^*V12*^ *dlg*^*RNAi*^ tumors. Co-expression of HA-tagged Ets21C (Ets21C^HA^) together with *Ras*^*V12*^ and *dlg*^*RNAi*^ did indeed increase tumor size (Fig. 1a,c,d). These results demonstrate that Ets21C is required for *Ras*^*V12*^ *dlg*^*RNAi*^ dependent tumor growth and also able to further stimulate tumor growth if provided in excess.

### The JNK pathway induces Ets21C expression

**Figure 2:**
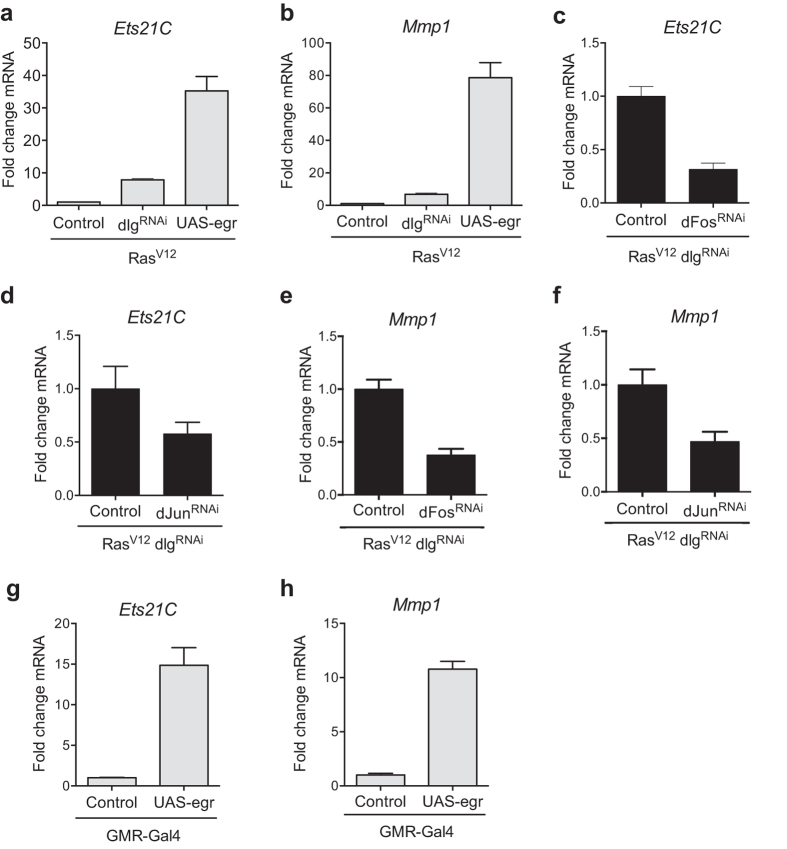
The JNK pathway regulates Ets21C expression. (**a,c,d**): qRT-PCR of *Ets21C* mRNA from dissected tumors with the indicated genotypes. (**b**,**e**,**f**): qRT-PCR for *Mmp1* mRNA from dissected tumors with the indicated genotypes. Ets21C is strongly upregulated in response to Egr (**a**) and this induction is blocked if Fos (**c**) or Jun (**d**) is removed. A similar response is observed for the known JNK pathway target *Mmp1* (**b**,**e**,**f**). (**g**,**h**) qRT-PCRs for *Ets21C* or *Mmp1* of eye discs that express Egr in the *GMR* expression domain indicating that the *Ets21C* and *Mmp1* genes are also activated by the JNK pathway in a non-tumor situation. Error bars indicate SD.

Our initial transcriptome profiles of *Ras*^*V12*^ *dlg*^*RNAi*^ tumors have revealed an upregulation of *Ets21C* (Toggweiler *et al*. in prep). Its expression is therefore likely to be regulated either by Ras^V12^ downstream signaling or the JNK pathway that is activated as a consequence of loss of polarity^9^. Previous studies on immune responses have shown that the expression of Ets21C depends on the JNK pathway^10^. This prompted us to test whether Ets21C expression was regulated by the JNK pathway in the *Ras*^*V12*^ *dlg*^*RNAi*^ tumor context as well (Fig. 2). Co-expression of *Ras*^*V12*^ and Eiger (Egr), a strong activator of the JNK pathway, resulted in a 30-fold induction of *Ets21C* expression in eye imaginal discs, a response even stronger than that observed in *Ras*^*V12*^ *dlg*^*RNAi*^ tumors (Fig. 2a). The known JNK pathway target *Mmp1* was upregulated to a similar extent (Fig. 2b). Complementary to these findings, a knockdown of the JNK transcription factors *jun* or *fos* decreased *Ets21C* as well as *Mmp1* expression (Fig. 2c–f). The regulation of *Ets21C* and *Mmp1* expression via Fos agrees with previous studies, however, Jun was thought to be dispensable for this process^11^. Nevertheless, our data suggest that Jun is at least partially involved in the transcriptional activation of *Ets21C* and *Mmp1*. In addition to the JNK pathway-dependent upregulation of *Ets21C* in a tumor background, we found that *Ets21C* and *Mmp1* are induced if we activate the JNK pathway in wild-type eye discs (Fig. 2g,h). This indicates that JNK activity is sufficient to induce *Ets21C* expression independent of elevated Ras activity or loss of polarity. As Ets21C levels are low in wild-type eye discs (data not shown), Ets21C might specifically be activated during stress situations and coordinate appropriate transcriptional responses. This corroborates previous findings of Ets21C being activated downstream of an immune response and a higher susceptibility of *Ets21C* mutant flies to pathogens^10^,^12^.

### Ets21C regulates the expression of JNK pathway targets

**Figure 3:**
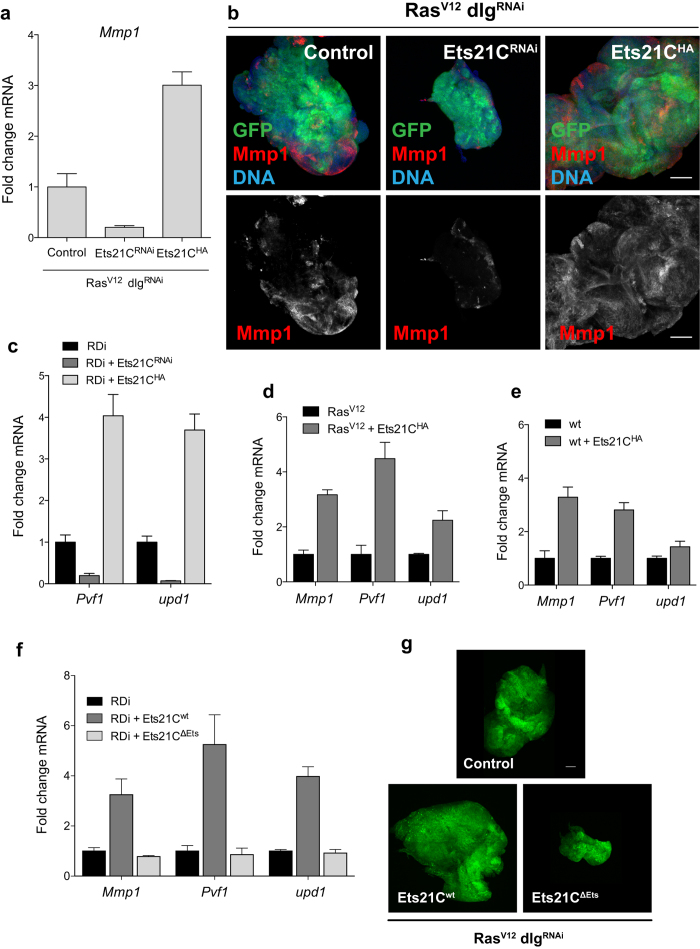
Ets21C influences the expression of JNK pathway targets. (**a**) Levels of *Mmp1* transcripts were reduced upon Ets21C depletion and increased if Ets21C was overexpressed in *Ras*^*V12*^ *dlg*^*RNAi*^ (*RDi*) tumors as measured by qRT-PCR. (**b**) Confocal images of *Ras*^*V12*^ *dlg*^*RNAi*^ tumors with the indicated genotypes. Tumor cells express GFP and are stained for Mmp1 (red) and DAPI (blue). Mmp1 protein levels change in a similar way as *Mmp1* transcripts upon Ets21C depletion or overexpression. (**c**) qRT-PCRs to detect *Pvf1* and *upd1* transcripts of dissected tumors with the indicated genotypes. *Pvf1* and *upd1* are differentially expressed upon changes in Ets21C levels. (**d,e**) *Mmp1*, *Pvf1* and *upd1* transcripts were also upregulated in *Ras*^*V12*^ tumors (**d**) or wild-type eye discs (**e**) overexpressing Ets21C. Error bars indicate SD. (**f**) The Ets21C protein variant lacking the DNA binding domain (Ets21C^ΔEts^) is no longer able to induce *Mmp1*, *Pvf1* and *upd1* expression. Error bars indicate SD. (**g**) Confocal images of control *Ras*^*V12*^ *dlg*^*RNAi*^ (*RDi*) tumors (upper panel) and tumors expressing full-length Ets21C (*Ets21C*^*wt*^) or the DNA binding mutant (*Ets21C*^*ΔEts*^) (lower panels). The upper and very left panels are composites of several images to display the whole tumor size. Scale bar: 100 μm.

Having established that *Ets21C* expression is activated via the JNK pathway, we sought to assess in more detail how Ets21C regulates tumor growth. For this purpose we established transcriptome profiles using microarrays of *Ras*^*V12*^ *dlg*^*RNAi*^ control tumors and *Ras*^*V12*^ *dlg*^*RNAi*^ tumors co-expressing either *Ets21C*^*RNAi*^ or *Ets21C*^*HA*^. Comparing control tumor transcriptomes to the profiles of tumors with higher or lower Ets21C levels revealed 22 genes that were significantly downregulated upon Ets21C depletion and upregulated if Ets21C was overexpressed (Supplementary Fig. 2a). Among these genes was *Mmp1*, a known JNK pathway target^8^. Depletion of Ets21C in *Ras*^*V12*^ *dlg*^*RNAi*^ tumors decreased *Mmp1* expression whereas overexpression of Ets21C increased *Mmp1* levels (Fig. 3a). In agreement with the qRT-PCR results, we found that Mmp1 expression is reduced at the protein level if Ets21C is depleted in *Ras*^*V12*^ *dlg*^*RNAi*^ tumors (Fig. 3b).

In addition to *Mmp1*, two further genes that are known to be downstream of JNK signaling were also found differentially expressed: *unpaired1* (*upd1*), and *PDGF and VEGF related factor 1*(*Pvf1*) (Supplementary Fig. 2a). *Upd1* encodes a cytokine that activates the JAK/STAT pathway. It has previously been found to be upregulated in *Ras*^*V12*^ *scrib*^*−/−*^ tumors in a JNK-dependent manner and to be essential for tumor growth^13^. Pvf1 is one of the *Drosophila* homologs of mammalian vascular endothelial growth factors (VEGFs) and platelet derived growth factors (PDGFs) and known to drive hemocyte proliferation in neoplastic tumors^14^. qRT-PCR analysis confirmed the differential expression of *upd1* and *Pvf1* upon altering Ets21C levels (Fig. 3c). Moreover, we found that other Upd- and PDGF/VEGF-like factors (Upd2, Upd3, Pvf2 and Pvf3) were also significantly upregulated in tumors overexpressing Ets21C (Supplementary Fig. 2b). Thus, our results show that Ets21C regulates the expression of targets of the JNK pathway, critical targets that explain, at least in part, the effects Ets21C exerts on tumor growth.

We next asked if the ability of Ets21C to induce *Mmp1*, *Pvf1* and *upd1* expression was dependent on the *Ras*^*V12*^ *dlg*^*RNAi*^ background or if it was a general function of Ets21C. In order to test this, we measured transcript levels of *Mmp1*, *Pvf1* and *upd1* in discs that co-expressed *Ras*^*V12*^ and *Ets21C*, and in wild-type eye discs overexpressing *Ets21C*. As in *Ras*^*V12*^ *dlg*^*RNAi*^ tumors, expression of Ets21C in *Ras*^*V12*^ tumors induced *Mmp1*, *Pvf1* and *upd1*, indicating that loss of polarity is not required for their activation (Fig. 3d). In agreement with these findings, *Ras*^*V12*^ *Ets21C* tumors grew to larger sizes and in three dimensions compared to *Ras*^*V12*^ tumors, which retain a rather hyperplastic morphology (Supplementary Fig. 2c). Furthermore, we also observed transcriptional activation of *Mmp1*, *Pvf1* and to a lesser extent *upd1* in wild-type eye discs that overexpress Ets21C, suggesting that Ets21C can activate these target genes in a physiological context (Fig. 3e). However, wild-type discs that overexpress Ets21C do not show an overgrowth phenotype (Supplementary Fig. 2d).

Having demonstrated that Ets21C can activate downstream targets of the JNK pathway that are necessary for neoplastic growth, we wanted to know if Ets21C would also regulate canonical JNK targets such as the phosphatase *puckered* (*puc*) or the apoptosis inducers *head involution defective* (*hid*) and *reaper* (*rpr*). To this end we monitored the expression levels of *puc*, *hid* and *rpr* with qRT-PCR in *Ras*^*V12*^ *dlg*^*RNAi*^ and *Ras*^*V12*^ tumors, as well as in wild-type discs, that overexpressed Ets21C and compared the transcript levels to the respective control tissue. We found that neither *Ras*^*V12*^ *dlg*^*RNAi*^ *Ets21C* nor *Ras*^*V12*^ *Ets21C* tumors substantially induce *puc*, *hid* or *rpr* expression (Supplementary Fig. 2f,g). Similarly, Ets21C expression in wild-type tissue did not affect *puc* or *hid* expression and only slightly increased *rpr* transcripts (Supplementary Fig. 2h).

### The ETS DNA binding domain is essential for Ets21C function

ETS proteins share as their unifying domain an ETS DNA binding motif, a variant of the winged helix-turn-helix motif that not only mediates binding to the DNA, but can also mediate protein-protein interactions^15^. In order to test if the Ets21C DNA domain was necessary for target gene activation, we generated a protein variant that lacks the ETS domain and tested its effects on *Ras*^*V12*^ *dlg*^*RNAi*^ tumors (Fig. 3f,g). Indeed, deleting the DNA binding domain (Ets21C^ΔEts^) abrogated the induction of the Ets21C targets *Mmp1*, *Pvf1* and *upd1* (Fig. 3f). Consistent with the effects on target gene expression, Ets21C^ΔEts^ was no longer able to enhance tumor growth as observed with full length Ets21C. It even caused a slight dominant-negative effect, as tumors expressing Ets21C^ΔEts^ were smaller than control tumors (Fig. 3g).

Taken together, our results demonstrate that the function of Ets21C critically depends on the DNA binding domain.

### Ets21C and the JNK pathway co-operatively induce specific target genes

**Figure 4:**
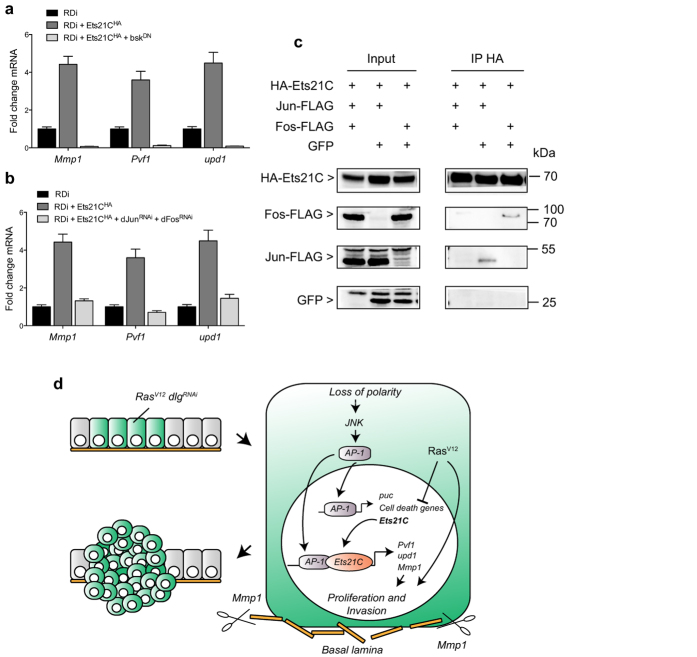
Ets21C cooperates with the JNK pathway to activate target genes. (**a**) Blocking the JNK pathway by expressing *bsk*^*DN*^ in *Ras*^*V12*^ *dlg*^*RNAi*^ (*RDi*) tumors that overexpress Ets21C abrogates the induction of *Mmp1*, *Pvf1* and *upd1* expression as assessed by qRT-PCR. (**b**) A similar effect is observed if the JNK pathway is blocked by knocking down *jun* and *fos*. Error bars indicate SD. (**c**) Ets21C physically interacts with Jun (second lane) or Fos (third lane). Kc167 cells were transfected with the transgenes indicated, together with *tub-Gal4* and *dTak1* to activate the JNK pathway. Anti-HA immunoprecipitates were immunoblotted first with anti-FLAG or anti-GFP antibodies (parts of the blot below 35 kDa). GFP was used as a negative control. After stripping, the same blot was incubated with anti-HA antibodies. (**d**) Model of Ets21C action: Ets21C is expressed in response to JNK pathway activation as a consequence of loss of polarity. Once Ets21C is expressed, it associates with AP-1 and together they drive the expression of target genes critical for tumor growth and invasion such as *Mmp1*, *Pvf1* and *upd1*.

Given the requirement of the DNA binding domain for Ets21C function and taking into account that the genes induced by Ets21C are known to be targets of the JNK pathway, we sought to clarify the relationship between Ets21C and JNK signaling regarding target gene induction. Based on our results, *Mmp1* is regulated by Ets21C and it is known to be a direct JNK pathway target as its expression is mediated by AP-1 binding sites in its regulatory region; we therefore hypothesized that Ets21C and AP-1 may co-operatively activate target gene expression^8^. To investigate this possibility, we first checked whether Ets21C outputs genetically depend on the JNK pathway. To this end, we co-expressed a dominant-negative form of Basket (Bsk^DN^), the *Drosophila* Jun kinase, together with *Ras*^*V12*^*, dlg*^*RNAi*^ and *Ets21C*^*HA*^ and monitored *Mmp1*, *Pvf1* and *upd1* expression. Blocking the JNK pathway abrogated expression of all three genes regardless of whether or not Ets21C^HA^ was overexpressed (Fig. 4a). This change was also reflected in a dramatic reduction of tumor size upon Bsk^DN^ expression (data not shown). We observed a similar effect when we inhibited JNK signaling at the level of the two AP-1 transcription factors, Jun and Fos: depletion of both together reverted the expression levels of *Mmp1*, *Pvf1* and *upd1* induced by Ets21C^HA^ to those found without Ets21C overexpression (Fig. 4b). These results show that Ets21C-dependent upregulation of *Mmp1*, *Pvf1* and *upd1* requires JNK signaling activity. Importantly, these results also show that *Pvf1* and *upd1* upregulation is not an indirect downstream consequence of JNK signaling mediated by Ets21C. Hence we conclude that Ets21C co-operates with AP-1 to mediate maximal target gene expression.

To gain further insight into the mechanism underlying this cooperation we performed co-immunoprecipitation experiments with Ets21C and/or Jun or Fos proteins to test for a physical interaction between the three proteins. We found that N-terminally HA-tagged Ets21C precipitates C-terminally FLAG-tagged Jun or Fos from *Drosophila* Kc167 cell lysates (Fig. 4c). While Jun and Fos were readily detectable, we did not find GFP in the precipitates, which shows the specificity of the interaction. Taken together, our genetic and biochemical data suggest that the function of Ets21C depends on a cooperation with AP-1.

Based on our data we propose a two-step model on how JNK signaling regulates tumor growth: in an early wave of pathway activity, AP-1 induces the expression of targets such as cell death genes, *puc*, and *Ets21C*. In a wild-type background this leads to apoptosis as demonstrated by numerous studies^16^,^17^,^18^,^19^. Neoplastic tumors, however, are protected against cell death through the presence of *Ras*^*V12*^, which allows the induction of a second tier of targets, such as *Mmp1*, *Pvf1* and *upd1*. Importantly, these targets not only depend on AP-1 activity, but also on Ets21C as their expression is reduced in tumors depleted for Ets21C (Fig. 4d). So far we can, however, not distinguish whether Ets21C only enhances and sustains AP-1 dependent target gene expression or whether it is required for their initial activation.

## Discussion

In this study, we identified the transcription factor Ets21C as a crucial regulator of tumor growth and demonstrate that its expression is activated by the JNK pathway. Moreover, we show that Ets21C regulates the expression of specific target genes that induce and sustain growth and invasiveness of *Ras*^*V12*^ *dlg*^*RNAi*^ tumors, possibly via a cooperation with AP-1.

The closest human orthologs of *Ets21C*^20^, *ETS-related gene* (*ERG*) and *Friend leukemia virus-induced erythroleukemia 1* (*FLI-1*), have also been linked to tumorigenesis. ERG is overexpressed in acute myeloid leukemia (AML) and is associated with a poor prognosis, whereas higher FLI-1 expression has been detected in triple negative breast cancer or in metastatic melanoma^21^,^22^. We show that Ets21C is not only sufficient to induce tumorigenesis, but required for tumor growth as a depletion of Ets21C strongly reduced tumor size. The smaller tumor size was accompanied by decreased levels of target genes known to drive tumor growth and malignancy, further underscoring the relevance of Ets21C. The finding that an *Ets21C* loss of function is blocking tumor growth so efficiently, suggests a more fundamental role for Ets21C in tumor growth than previously assumed. Kulshammer *et al*.^11^ have also tested an *Ets21C*^*RNAi*^ in *Ras*^*V12*^*, scrib*^*−/−*^ tumors, but were not able to observe any remarkable effects besides a partial rescue of the pupariation delay that is commonly associated with neoplastic tumor growth in flies. An explanation for this discrepancy might simply be different strengths of the RNAi lines used.

Loss of epithelial polarity caused by a depletion of *dlg* activates the JNK pathway that critically affects tumor growth. In agreement with previous studies, we found elevated *Ets21C* transcript levels in *Ras*^*V12*^ *dlg*^*RNAi*^ tumors and show that JNK signaling is required for the upregulation^10^,^11^,^23^. The JNK pathway activates the AP-1 transcription factor, which in its prototypical form is a dimer consisting of Jun and Fos proteins. In the *Ras*^*V12*^*, scrib*^*−/−*^ context, Fos has been described as the main effector of the JNK pathway, as a depletion diminishes tumor growth and abrogates induction of target genes, while no such effect have been observed for Jun^11^. However, our data point towards at least a partial role for Jun activating target genes, since a knockdown of Jun reduced expression of *Ets21C* and *Mmp1*.

Given the strong effects that Ets21C exerts on tumor growth, we sought to identify Ets21C dependent target genes that could explain this phenomenon. Transcriptional profiles revealed an upregulation of *Mmp1*, *Pvf1* and *upd1* in tumors that overexpressed Ets21C and downregulated in tumors depleted of Ets21C (Fig. 3 and Supplementary Fig. 2). *Mmp1, Pvf1 and upd1* have previously been shown to be induced by the JNK pathway and to be essential for tumor growth and invasion^8^,^13^,^14^. Besides *upd1*, also *upd2* and *upd3* have been reported to be induced in neoplastic tumors in a JNK pathway dependent manner^11^,^13^,^23^. In agreement with these reports we observed an induction of *upd2* and *upd3* in *Ras*^*V12*^ *dlg*^*RNAi*^ *Ets21C* tumors, but to a lesser extent than *upd1* (Supplementary Fig. 2b). This might on the one hand depend on the use of *dlg* instead of *scrib* as Bunker *et al*. find a stronger activation of Upd cytokines in *scrib* mutant wing discs compared to *dlg* mutants. On the other hand, additional factors might influence transcriptional activation such as the presence of *Ras*^*V12*^, the exact timing of sample collection or the genetic system used. Furthermore, the strength of induction might not necessarily correlate with the effect on tumor growth. Although *upd3* exhibits the strongest upregulation in neoplastic tumors, it has been shown that co-expression of Upd1 and Upd2 together with Ras^V12^ leads to much larger tumors than the combination of Ras^V12^ and Upd3^13^.

While Ets21C is able to stimulate the expression of the JNK downstream targets *Mmp1*, *Pvf1* and *upd1*, we did not observe an obvious regulation of canonical JNK pathway targets such as the phosphatase *puc* or the apoptosis inducers *hid* or *rpr* (Supplementary Fig. 2f–i), suggesting that Ets21C only regulates a specific set of JNK pathway targets. In contrast, a previous study has described a slight upregulation of *puc* in *Ras*^*V12*^ *Ets21C* tumors^11^. Trivial differences such as when the samples were collected, transgene strength, or genetic background could account for this difference. For example, an elevation of *puc* expression could also originate from an indirect increase in JNK activity due to stresses in an older, larger tumor. These results are entirely consistent with our model that Ets21C can activate certain JNK targets in a context dependent manner.

Finally, we wondered if and how Ets21C regulates transcriptional outputs of the JNK pathway (Fig. 3). (1) Ets21C could interact with Jun and/or Fos. (2) Ets21C could activate or interact with an unknown factor that feeds back on the JNK pathway. (3) Ets21C could use a combination of both (1) and (2). We show genetically that in *Ras*^*V12*^ *dlg*^*RNAi*^ tumors the effects of Ets21C, both phenotypically and on a target genes level, fully depend on an active JNK pathway. If the latter is blocked, for example, by co-expressing Bsk^DN^ with *Ras*^*V12*^ *dlg*^*RNAi*^ and *Ets21C*^*HA*^, tumors remain small and there is no induction of *Mmp1*, *Pvf1* or *upd1*. These results support all possibilities (1)–(3), but exclude an autonomous function of Ets21C. A physical interaction between Ets21C and Jun and Fos has previously been proposed based on large scale mass-spectroscopy data^24^. Here, we show that HA-tagged Ets21C does indeed bind FLAG-tagged Jun or Fos, showing that the proteins could interact physically to regulate target gene expression. The binding is consistent with studies in mammalian systems that have shown a physical interaction between AP-1 and ETS proteins including ERG, the mammalian homolog of Ets21C^25^,^26^,^27^. In agreement with a cooperative transcriptional activation, we found Ets21C and AP-1 binding sites co-occurring in the putative regulatory regions of the analyzed target genes^28^. We therefore think that Ets21C likely activates target genes via a cooperation with AP-1. However, additional factors that are activated by the JNK pathway might contribute to target gene activation as well and could explain why Ets21C activates certain JNK downstream target genes and others not.

In summary, we show that Ets21C fulfills a crucial role in the regulation of neoplastic tumor growth as a loss of function critically reduced tumor growth, whereas an excess of Ets21C further increased tumor size. While Ets21C was previously accredited only with a role in fine-tuning the transcriptional program of neoplastic tumors^11^, our results point towards a more fundamental role as activator of a specific set of target genes that drive tumor growth and invasion (see Fig. 4d).

## Materials and Methods

### Fly stocks and genetics

To test for an effect on tumor growth, UAS-RNAi lines or UAS-cDNAs were crossed to females of the following tester fly stock: *eyFlp; UAS-Ras*^*V12*^*, UAS-dlg*^*RNAi*(*VDRC41134*)^*, UAS-GFP/CyO, tub-Gal80*^*BL9491*^*; act* > *CD2* > *Gal4, UAS-GFP*^*S65T*^. To express the transgenes in a non-tumor background, the following flies were used: *eyFlp; Sp/CyO; act* > *CD2* > *Gal4, UAS-GFP*^*S65T*^. To generate tumors that express *Ras*^*V12*^ only, we used: *eyFlp; act* > *y*^*+*^ > *Gal4, UAS-GFP/CyO, tub-Gal80*^*BL9491*^*; UAS-Ras*^*V12*^*/TM6b* flies. *yw* flies were used for control crosses. To generate MARCM clones, *eyFlp; FRT40, tub-Gal80/SM5; tub-Gal4, UAS-GFP/TM6b* females were crossed to *yw; FRT40; MKRS/TM6b* or *yw; FRT40, ΔEts21C*^*21*^*/SM5;* +*/TM6b* or *yw; FRT40; UAS-Ras*^*V12*^*, UAS-dlg*^*RNAi*(*BL34854*)^*/TM6b* or *yw; FRT40, ΔEts21C*^*21*^*/SM5; UAS-Ras*^*V12*^*, UAS-dlg*^*RNAi*(*BL34854*)^*/TM6b* males. Flies and larvae were reared and kept at 25 °C. Other RNAi lines from the Vienna Drosophila RNAi library (VDRC) used were: *UAS-Ets21C*^*RNAi* (*51225*)^, *UAS-dJun*^*RNAi*(*107997*)^. From the DGRC Trip^RNAi^: *UAS-Fos*^*RNAi*(*BL33379*)^. The following fly stocks used for overexpression experiments were from FlyORF: *UAS-Ets21C*^*HA*(*F000624*)^. Other fly stocks used were: *UAS-egr*16, *GMR-Gal4*^29^, *UAS-bsk*^*DN*^.

### Quantification of tumor volume

Tumor bearing larvae were dissected 6–7 days after egg laying (AEL). For confocal imaging and quantifications, larvae were fixed according to standard protocols and tumors were mounted in VectaShield (Vector Laboratories). To minimize squeezing of tumors by the cover slip, one layer of double scotch tape was placed between cover slip and glass slide. Image stacks were thresholded for GFP and analyzed with Icy to calculate the tumor volume^30^.

### Cloning and generation of transgenes

#### Truncated proteins

A plasmid with the open-reading frame of the *Ets21C* A isoform was obtained from FlyORF. To produce the Ets21C protein variant, an overlapping PCR was performed to delete aminoacids 254–339 (ΔEts), which were annotated as the ETS domain according to a SMART alignment^31^. The truncated protein version was cloned into pUAST.attB via Acc65I and XbaI cut sites that had been attached in the PCR. With the same cloning strategy, a wild-type version of Ets21C without HA-tag was cloned as well to exclude any adverse effects of the HA tag. Both constructs were inserted via the ΦC31 integrase system into landing site *ZH-86Fb*^32^.

#### Expression vectors for cell culture

*fos* or *Ets21C* cDNA was amplified by PCR and inserted into the triple HA-containing vector pMZ55 via NheI and XbaI sites. The fragments were then subcloned along with the HA-tag into pUAST via Acc65I and XbaI sites.

### Generation of the *Ets21C* deletion

*Ets21C* was removed with an *in trans* recombination of flanking PiggyBac (PBac) elements according to the strategy of Parks *et al*.^33^. PBac elements *WH Ets21C*^*f03639*^ and *RB rempA*^*e02928*^ were combined with *hsp70-flp* on the X-chromosome and then brought together in trans. *hsFlp; Ets21C*^*f03639*^*/rempA*^*e02928*^ progeny were heat-shocked two days AEL for 1 h at 37 °C. The heat-shock was repeated the next two days. Single adult males were crossed to balancer stocks and the progeny was tested by PCR for recombination with one primer binding to the remaining part of *WH Ets21C*^*f03639*^ going in direction of the RB element and another primer binding to the flanking region of the RB element going in direction of the WH element. A product is only yielded if recombination was successful as the region between the primer binding sites is otherwise too long. The recombination also removed a part of the neighboring gene *rempA* (*CG11838*). However, mutant clones did not show any obvious phenotypes (Supplementary Fig. 1a,b) and expression of a RNAi targeting *rempA* in *Ras*^*V12*^ *dlg*^*RNAi*^ cells did not affect tumor growth (data not shown).

### Immunofluorescence

Fixation and immunostainings of eye imaginal discs and tumors were performed according to standard protocols. Primary antibodies used in this study were: anti-Mmp1 (1/200, 3B8D12, Developmental studies hybridoma bank (DSHB), Iowa City, IO, USA) and anti-DE-cadherin (1/200, DCAD2, DSHB). Alexa fluor 594 (1/500, Molecular Probes, Eugene, OR, USA) was used as label for the secondary antibody. Imaginal discs were mounted in VectaShield (Vector Laboratories, Burlingame, CA, USA). Images were taken with a Zeiss Lsm710 confocal microscope (Zeiss, Oberkochen, Germany) and processed with ImageJ and Adobe Photoshop.

### RNA isolation and real-time PCR

For RNA isolations, larvae were dissected in Ringers plus 0.05% Tween-20 and tumors were collected in an 1.5 ml Eppendorf tube. Tumors were centrifuged for 10 min at 4 °C and pellets were snap freezed in liquid nitrogen, stored at −80 °C or processed further. For each experiment total RNA was isolated from 30 tumors or 45 wild-type eye discs with the indicated genotypes using the NucleoSpin RNA kit (Machery Nagel, Düren, Germany). Following an additional Dnase digest for 1 h at 37 °C (DNA-free^TM^ kit, Invitrogen, Thermo Fisher Scientific,Waltham, MA, USA), 500 ng of total RNA was used for cDNA synthesis with the transcriptor High Fidelity cDNA Synthesis Kit (Roche, Basel, Switzerland) using oligo-dT primers. Quantitative PCR reactions were performed in triplicates using the MESA Green qPCR Mastermix Plus for SYBR Assay (Eurogentec, Liège, Belgium) and an ABI Prism SDS 7900 HT (Applied Biosystems, Foster City, CA, USA). All measurements of transcript levels were normalized to *actin-5C*, *alpha-tubulin* and *TATA box binding protein* (*TBP*). One representative assay is shown in the results and error bars represent standard deviations of the technical replica. Primers were designed with Roche Universal Probe Library or with Primer3plus. Whenever possible an intron-spanning assay was chosen. Primers used were: *Ets21C*: F: caacgacgacgaaccaaat, R: gttcgcgttggacgaatc, *Mmp1*: F: gaaggctcggacaacgagt, R: gtcgttggactggtgatcg, *Pvf1*: F: aagccggaacaccattgac, R: catgatgctgcgcttaaagt, *upd*: F: gcacactgatttcgatacgg, R: ctgccgtggtgctgtttt, *actin*: F: gcccatctacgagggttatgc, R: aatcgcgaccagccagatc, *puc*: F: gccacatcagaacatcaagc, R: ccgttttccgtgcatctt, *hid*: F: tctacgagtgggtcaggatgt, R: gcggatactggaagatttgc, *rpr*: F: tcgatttctactgcagtcaagg, R: gagtaaactaaaattgggtgggtgt, *alpha-tubulin at 84C*: F: gccagatgccgtctgacaa, R: agtctcgctgaagaaggtgttga, *TATA binding protein*: F: cgcgcatcatccaaaagc, R: gccgaccatgttttgaatcttaa.

### Microarrays

Total RNA was isolated and Dnase digested as described above from tumors with the following genotypes: *eyFlp; UAS-Ras*^*V12*^*, UAS-dlg*^*RNAi*^*/*+*; act* < *CD2* < *Gal4, UAS-GFP/*+ or *eyFlp; UAS-Ras*^*V12*^*, UAS-dlg*^*RNAi*^*/UAS-Ets21C*^*RNAi*^*; act* < *CD2* < *Gal4, UAS-GFP/*+ or *eyFlp; UAS-Ras*^*V12*^*, UAS-dlg*^*RNAi*^*/Sp or CyO; act* < *CD2* < *Gal4, UAS-GFP/UAS-Ets21C*^*HA*^. RNA was sent to the Genomics platform in Geneva (http://www.ige3.unige.ch/genomics-platform.php) for further processing and hybridization to Affymetrix GeneChip arrays for *Drosophila* (Affymetrix, Santa Clara, CA, USA). Microarrays were done from three biological replica. Bioinformatic and statistical analysis was also done in Geneva. Briefly, expression values were normalized with the RMA algorithm and statistical analysis was done with a 1-way ANOVA with constrasts and multiple test corrections (false discovery rate (FDR) Benjamini & Hochberg, 2005). Criteria for filtering were a p-value with FDR correction ≤0.05 and a fold change ≥2. Microarray data are available in the ArrayExpress database (www.ebi.ac.uk/arrayexpress) under the accession number E-MTAB-4315.

### *Drosophila* cell culture and transfections

Kc167 cells were cultured in M3 + PYRE medium supplemented with 5% FBS and 1% penicillin/streptomycin at 25 °C. Cells were transfected with expression vectors using FuGene®HD (Promega, Madison, WI, USA) according to the manufacturers protocol.

### Immunoprecipitation and western blotting

Kc167 cells (2 × 10^6^ cells per well) were seeded into a 6-well plate and transfected with the indicated expression vectors. *UAS-GFP* was used as control for transfection efficiency and to keep the total amount of DNA transfected constant. Cells were harvested 48 h post-transfection and lysed in lysis buffer containing 20 mM Tris-HCl pH 7.5, 1.5 mM MgCl_2_, 75 mM NaCl, 1 mM EGTA, 5% Glycerol, 0.25% Nonidet P-40 and protease inhibitors (Complete Mini, Roche). Following 5 min of sonication with a Bioruptor, the cell suspension was centrifuged at full speed at 4 °C and supernatants were mixed with 30 μl of Protein A sepharose beads (GE Healthcare, UK) and 1 μg of HA antibody (sc-805, Santa Cruz Biotechnology, Santa Cruz, CA, USA) and allowed to rotate for 4 h at 4 °C. Beads were collected (2500 × g, 3 min at 4 °C), washed 2× with lysis buffer and stored at −20 °C until further processing.

For western blot analysis, protein complexes were eluted from the beads, denatured and resolved on 4–12% NuPAGE Bis-Tris gels (Invitrogen, Thermo Fisher Scientific) and transferred to a PVDF membrane (Amersham Hybond-P, GE Healthcare, UK). After blocking with 5% milk, the membrane was incubated with either anti-FLAG M2 (mouse, 1/2000, Sigma-Aldrich, St. Louis, MO, USA) or anti-GFP (MAB3580, 1/2000, Millipore, Billerica, MA, USA) antibodies followed by secondary goat anti-mouse antibodies conjugated with horseradish peroxidase (1/5000, Jackson Laboratories, West Grove, PA, USA). Signals were detected with Western Bright Quantum detection reagent (Advansta, Menlo Park, CA, USA). After stripping (Re-Blot Plus Strong Solution, Millipore), membranes were blocked and incubated with anti-HA (mouse, 1/2000, HA.11, Covance, Princeton, NJ, USA) antibody.

## Additional Information

**How to cite this article**: Toggweiler, J. *et al*. The transcription factor Ets21C drives tumor growth by cooperating with AP-1. *Sci. Rep.* **6**, 34725; doi: 10.1038/srep34725 (2016).

## Supplementary Material

Supplementary Information
