# Supplementary material for: The transcription factor Ets21C drives tumor growth by cooperating with AP-1
Source: Sci Rep. 2016 Oct 7;6:34725. doi: 10.1038/srep34725 (PMC5054425; doi:10.1038/srep34725)
Supplement: Supplementary Information [file srep34725-s1.pdf]

The transcription factor Ets21C drives tumor growth by cooperating with AP-1

Janine Toggweiler[1], Maria Willecke[1, 2] and Konrad Basler[1*]

[1] Institute of Molecular Life Sciences, University of Zurich, Zurich, Switzerland

[2] Present address: Neural Control of Movement Laboratory, Department of Health Sciences and Technology, ETH Zurich, Zurich, Switzerland

Supplementary Information

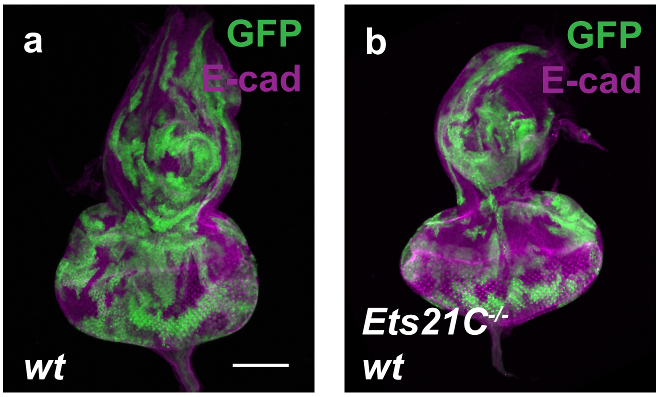

Supplementary Figure 1: A loss of *Ets21C* does not affect normal cells

(a) and (b) Discs contain *ey-FLP* induced MARCM clones [34] that are positively labeled with GFP (green). Additionally, discs are stained for E-cadherin (E-cad) to visualize cell outlines (magenta). (a) Wild-type clones, (b) Clones homozygous mutant for *Ets21C*. Scale bar: 100µm.

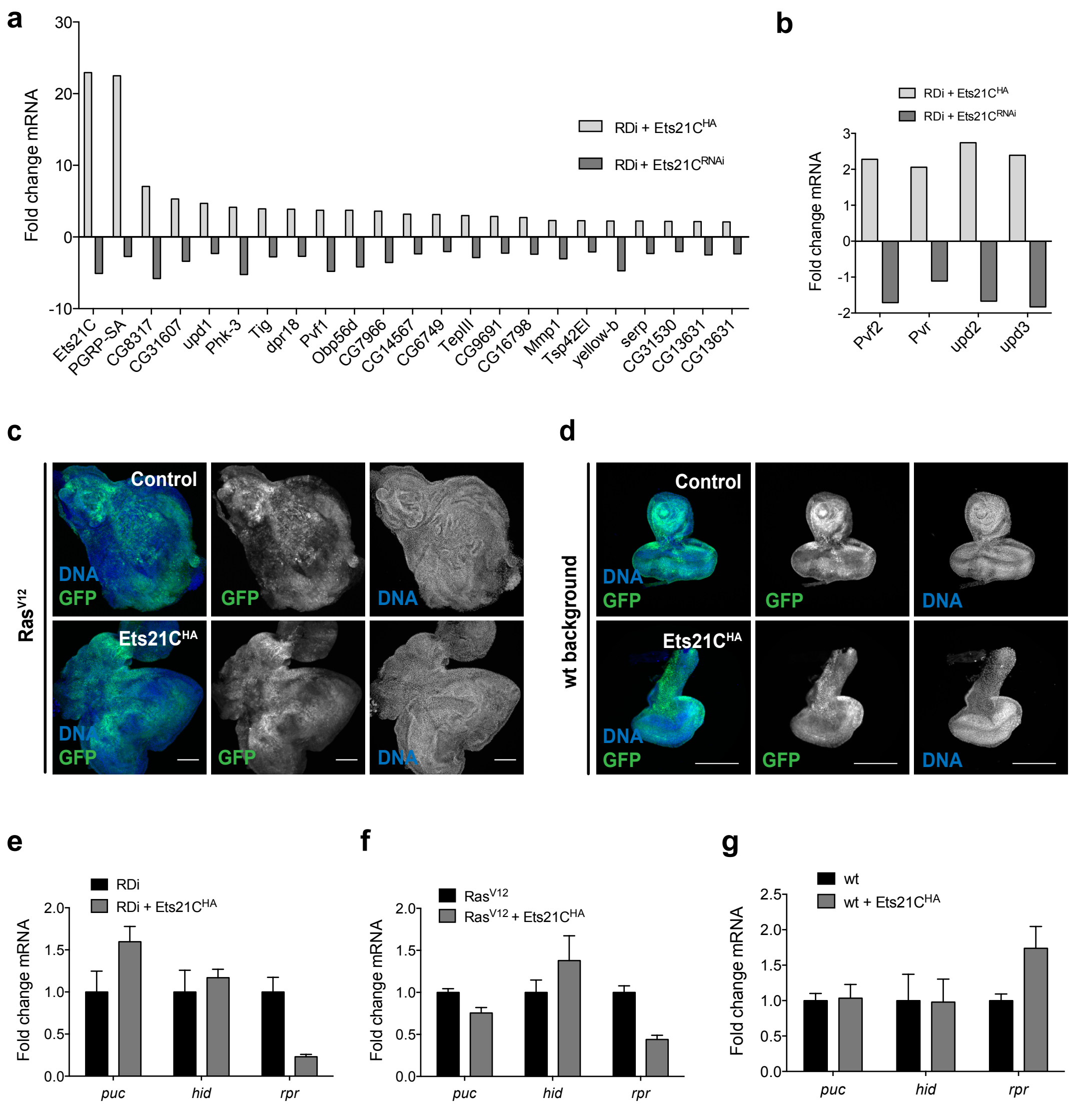

Supplementary Figure 2: Gene expression changes upon Ets21C overexpression or depletion

(a) 22 genes that are significantly (fold change ≥2) upregulated upon Ets21C overexpression and downregulated with *Ets21C*^{RNAi} in *Ras*^{V12} *dlg*^{RNAi} tumors (*RDi*). (b) Differential expression of additional *Pvfs* and *upd* genes upon changes in *Ets21C* levels. (c) Confocal images of control *Ras*^{V12} tumors (upper) or *Ras*^{V12} tumors expressing Ets21C (lower). Scale bar: 100µm. (d) Confocal images of wild-type eye discs (upper) or eye discs overexpressing Ets21C (lower). Scale bar: 200µm. (e) - (g)

qRT-PCRs to detect *puc*, *hid* or *rpr* transcripts of dissected tumors (e) and (f) or wild-type eye discs (g) with the indicated genotypes. *puc*, *hid* or *rpr* expression did not change substantially upon Ets21C overexpression. Error bars indicate SD.
